# Idiopathic pulmonary fibrosis

**DOI:** 10.1186/1750-1172-3-8

**Published:** 2008-03-26

**Authors:** Eric B Meltzer, Paul W Noble

**Affiliations:** 1 Department of Medicine, Division of Pulmonary, Allergy and Critical Care, Duke University Medical Center, Durham, North Carolina 27710, USA

## Abstract

Idiopathic pulmonary fibrosis (IPF) is a non-neoplastic pulmonary disease that is characterized by the formation of scar tissue within the lungs in the absence of any known provocation. IPF is a rare disease which affects approximately 5 million persons worldwide. The prevalence is estimated to be slightly greater in men (20.2/100,000) than in women (13.2/100,000). The mean age at presentation is 66 years. IPF initially manifests with symptoms of exercise-induced breathless and dry coughing. Auscultation of the lungs reveals early inspiratory crackles, predominantly located in the lower posterior lung zones upon physical exam. Clubbing is found in approximately 50% of IPF patients. Cor pulmonale develops in association with end-stage disease. In that case, classic signs of right heart failure may be present. Etiology remains incompletely understood. Some environmental factors may be associated with IPF (cigarette smoking, exposure to silica and livestock). IPF is recognized on high-resolution computed tomography by peripheral, subpleural lower lobe reticular opacities in association with subpleural honeycomb changes. IPF is associated with a pathological lesion known as usual interstitial pneumonia (UIP). The UIP pattern consists of normal lung alternating with patches of dense fibrosis, taking the form of collagen sheets. The diagnosis of IPF requires correlation of the clinical setting with radiographic images and a lung biopsy. In the absence of lung biopsy, the diagnosis of IPF can be made by defined clinical criteria that were published in guidelines endorsed by several professional societies. Differential diagnosis includes other idiopathic interstitial pneumonia, connective tissue diseases (systemic sclerosis, polymyositis, rheumatoid arthritis), *forme fruste *of autoimmune disorders, chronic hypersensitivity pneumonitis and other environmental (sometimes occupational) exposures. IPF is typically progressive and leads to significant disability. The median survival is 2 to 5 years from the time of diagnosis. Medical therapy is ineffective in the treatment of IPF. New molecular therapeutic targets have been identified and several clinical trials are investigating the efficacy of novel medication. Meanwhile, pulmonary transplantation remains a viable option for patients with IPF. It is expected that, during the next decade, considerable progress will be made toward the understanding and treatment of this devastating illness.

## Disease name and synonyms

Idiopathic pulmonary fibrosis (IPF).

Synonym: cryptogenic fibrosing alveolitis (CFA) was the preferred term in Europe until terminology was simplified by consensus conference [1].

## Definition

Idiopathic pulmonary fibrosis (IPF) is a chronic disease that manifests over several years and is characterized by scar tissue within the lungs, in the absence of known provocation. Exercise-induced breathlessness and chronic dry cough are the prominent symptoms.

IPF belongs to a family of lung disorders known as the interstitial lung diseases (ILD) or, more accurately, the diffuse parenchymal lung diseases (DPLD). Within this broad category of diffuse lung diseases, IPF belongs to the subgroup known as idiopathic interstitial pneumonia (IIP). By definition, the etiology of IIP is unknown. There are seven distinct IIPs, differentiated by specific clinical features and pathological patterns [2]. IPF is the most common form of IIP. It is associated with the pathologic pattern known as usual interstitial pneumonia (UIP); for that reason, IPF is often referred to as IPF/UIP. IPF is usually fatal, with an average survival of approximately three years from the time of diagnosis [3-5]. Older studies suggested that five-year mortality for IPF was only 50%, but this estimate was derived prior to the recognition of nonspecific interstitial pneumonia (NSIP), a pathological subtype of IIP that mimics IPF in its clinical presentation [6-8]. NSIP has a more favorable prognosis and the almost certain inclusion of NSIP cases in older studies of IPF mortality accounts for differences in observed outcome [9]. By definition, IPF/UIP must be discriminated from NSIP.

## Epidemiology

The incidence and prevalence of IPF are difficult to determine because uniform diagnostic criteria have only recently been defined [1]. Historical information relating to vital statistics relied on population studies which utilized diagnostic coding data and death certificates to identify cases. The accuracy of this information can be questioned, especially when studies were performed in the era of undefined diagnostic criteria.

The best available data suggests an incidence of approximately 10.7 per 100,000 persons for men; and 7.4 per 100,000 persons for women. The prevalence of IPF is slightly greater at 20.2 men per 100,000 and 13.2 women per 100,000 [10,11]. Data from around the world demonstrates that IPF favors no particular race, ethnic group or social environment. It is estimated that IPF affects at least 5 million persons worldwide. It also appears that, during the last decade, the incidence of IPF was on the rise [12].

Cigarette smoking is strongly associated with IPF. One study reported a correlation between smoking history (20–40 pack-years) and risk for IPF, with an odds ratio of 2.3 (95% confidence interval, 1.3 to 3.8) for smokers [13].

IPF affects men more than women. In addition, the incidence of IPF increases with age. IPF most commonly appears between the fifth and seventh decades of life, with two-thirds of all cases arising in patients over 60 years of age [1]. The mean age at presentation is 66 years old [1]. IPF occurs infrequently in those younger than 40 and rarely affects children, if at all. A large U.S. population-based study noted a significant difference in prevalence by age [10]. This study found that the prevalence of IPF was only 2.7 cases per 100,000 amongst those aged 35 to 44 years-old; meanwhile, 175 cases per 100,000 were found among persons over the age of 75 years.

Familial cohorts of IPF are described in dozens of reports, though sporadic cases constitute the majority of disease. Clinical features of familial IPF are indistinguishable from those of the sporadic form, excepting for an earlier age of onset [14]. Familial IPF is defined as two or more verified cases within a group of relatives belonging to a primary family unit (parents, children and siblings). Familial IPF accounts for 0.5 to 2% of all cases of IPF. Lung inflammation has been identified in unaffected members of families with IPF [15]. The largest description of familial pulmonary fibrosis identified 111 families with 309 affected family members [16]. This study identified an autosomal dominant vertical transmission pattern. Also described were families in which more than one variant of idiopathic interstitial pneumonia was present. This finding in particular suggests that pulmonary fibrosis, independent of specific form, is a common endpoint for genetically-mediated disease-forming pathways. While single gene defects have yet to be identified, a recent and intriguing report described polymorphisms of *hTERT *and *hTR *in a cohort of patients with familial IPF [17]. These two genes are involved in the regulation of telomere length and thereby play a pivotal role in controlling cell death and aging.

## Clinical description

### History and physical

IPF patients experience breathlessness upon exertion. They are often bothered by a dry cough which interferes with daily activities. The onset of symptoms is slow, but symptoms become progressively worse over time. The initial presentation of breathlessness is commonly attributed to aging, cardiac disease, or emphysema which results in typical delays of diagnosis. Retrospective analysis of IPF patients suggests that symptoms precede diagnosis by a period of 6 months to 2 years [18]. Symptoms such as weight loss, fever, and arthralgias are unusual in IPF and should prompt an investigation for secondary causes of pulmonary fibrosis. Gastro-esophageal acid reflux is present in close to 90% of patients with IPF but often occurs without symptoms [19].

Auscultation of the lungs reveals early inspiratory crackles, predominantly located in the lower posterior lung zones upon physical exam. These rales have a fine acoustic character reminiscent of the sound made by *Velcro*.

Clubbing is found in approximately 50% of patients with IPF. There are no other physical manifestations, unless cor pulmonale has developed in association with end-stage disease. In that case, classic signs of right heart failure may be present.

The examination of patients with IPF should attempt to identify those signs suggesting an alternative diagnosis such as systemic sclerosis or polymyositis that can be associated with secondary pulmonary fibrosis. To that end, the examiner should look for sclerodactily, scleroderma, proximal muscle weakness and telangiectasias. The history should exclude Raynaud's phenomenon.

### Diagnostic laboratory findings

There are no specific laboratory abnormalities in IPF. However, mild elevation of the erythrocyte sedimentation rate, a low-positive titer of anti-nuclear antibody (ANA) and/or low-positive rheumatoid factor can be seen and are thought to represent a general state of inflammation. In advanced disease, blood counts may reveal polycythemia.

A high titer of autoantibodies suggests an alternative diagnosis such as connective tissue disease. In order to exclude secondary causes of pulmonary fibrosis, a broad panel of autoantibodies should be ordered during the initial evaluation.

### Physiologic changes

Routine spirometry reveals decreased measures of forced vital capacity (FVC) and forced expiratory volume in one second (FEV_1_). The ratio of FEV_1_/FVC remains normal (or increased) in IPF, consistent with restrictive physiology. Lung volume measurements confirm restrictive physiology, usually manifest in reduction of total lung capacity (TLC). Restrictive physiology is the consequence of reduced pulmonary compliance. Changes in compliance can be attributed to the accumulation of parenchymal scar tissue and the subsequent distortion of normal lung architecture.

Gas exchange is impaired in IPF which can be demonstrated by measurement of the diffusion capacity. Declining diffusion capacity can sometimes precede changes in lung volume. Isolated impairment of diffusion capacity can be found during the early stages of IPF.

The resting arterial blood gas is usually normal. Mild hypoxemia and mild respiratory alkalosis can occur in end-stage disease. Although resting arterial oxygen saturation remains normal, oxygen desaturation is commonly found during exercise. The main cause for exercise-induced hypoxemia is ventilation-perfusion (V/Q) mismatching, as opposed to anatomic shunting or reduced diffusion capacity [20].

### Natural history and prognosis

The natural history of IPF is incompletely known. IPF usually assumes a course of relentless physiologic deterioration. However, some patients remain stable for extended periods and individual outcomes can be highly variable [18]. Still, long-term survival with biopsy proven IPF is not expected. The median survival time demonstrated in recent studies using the modern definition of IPF is between 2 and 5 years, counting from the time of diagnosis [3-5,9].

New insight into the natural history of IPF has been gleaned from secondary analysis of the placebo groups assembled for recent multi-center clinical trials [21-23]. It appears that three potential clinical courses exist: a) slowly progressive disease (the most common); b) disease marked by episodic acute exacerbations; and c) rapidly progressive disease [18]. At present, there are no means for accurately predicting the clinical course. Nevertheless, acute exacerbations deserve special attention.

### Acute exacerbations of IPF

Japanese physicians were the first to describe acute, unexpected deterioration in patients with IPF. This phenomenon has been called the "acute exacerbation" or, more euphemistically, the "terminal complication" of IPF [24,25]. Acute exacerbations in IPF (AE-IPF) are characterized by sudden worsening of respiratory symptoms accompanied by hypoxemia and the appearance of new radiographic infiltrates. It is important to clinically distinguish AE-IPF from pulmonary embolism, congestive heart failure, pneumothorax and infection. Acute exacerbations can occur in patients with known IPF, but AE-IPF also presents as the initial manifestation of IPF [26]. The yearly incidence of AE-IPF is between 10 and 15% of all patients who are at risk. This estimate is based on data derived from the placebo-control groups of two large randomized clinical trials [21,27]. AE-IPF is recognized by clinical criteria established in the first published case series from Japan [24]. The diagnosis, in the setting of known IPF, requires all of the following: a) acute worsening of dyspnea within the last month; b) deterioration from baseline of either vital capacity or gas exchange (usually documented by a wide A-a gradient); c) new radiographic infiltrates; and d) the absence of alternative causes for clinical deterioration [28]. The development of AE-IPF in patients without known IPF can be recognized when consensus criteria for IPF (see below) are satisfied and the patient presents with acute respiratory failure. The radiographic features of AE-IPF are ground glass opacification superimposed on typical interstitial markings. The histopathology of AE-IPF can demonstrate usual interstitial pneumonia (UIP; see below) with superimposed diffuse alveolar damage (DAD), characterized by alveolar epithelial injury and hyaline membranes. An alternate histopathology of AE-IPF consists of UIP with superimposed organizing pneumonia [26]. The prognosis of AE-IPF is poor. Several small series of AE-IPF report that the mortality of this condition ranges between 78 to 96% [29-31]. However, this data is skewed by methods that used autopsy results for case finding. Still, an association is reported between the need for mechanical ventilation and mortality in AE-IPF. The usual approach to treating AE-IPF is to use corticosteroids but the benefit of such has not been established.

### Pulmonary hypertension and IPF

Pulmonary hypertension has been reported to occur in 32 to 84% of patients with IPF. The exact prevalence remains unclear because triggers for the evaluation of pulmonary pressures and the best method to detect pulmonary hypertension in IPF remain unsettled [32]. Diffusion capacity is strongly correlated with pulmonary hypertension, being inversely related [33,34]. However, the severity of restrictive physiology has little bearing on the prevalence or degree of pulmonary hypertension. Several studies have demonstrated a lack of correlation between pulmonary artery pressure and forced vital capacity (FVC) [33,35]. Right-heart catheterization is the best diagnostic test for pulmonary hypertension but the implementation of such invasive testing is difficult to justify in the absence of data demonstrating a benefit to treatment of pulmonary hypertension in IPF. Still it is clear that the presence of pulmonary hypertension in IPF adversely affects survival [33,36].

### IPF and emphysema

Several groups have described a syndrome in which IPF coexists with pulmonary emphysema [37-39]. This comes as no surprise since both diseases are associated with a history of exposure to cigarette smoke. Combined IPF and emphysema is characterized by upper lobe emphysema and lower lobe fibrosis. Physiologic testing of these patients reveals preserved lung volume indices contrasted by markedly impaired diffusion capacity. The incidence of combined IPF and emphysema remains unknown but smaller case series suggest that this subgroup may comprise up to 35% of patients with IPF [38].

Combined IPF and emphysema is a strong determinant of secondary pulmonary hypertension [39]. In addition, combined IPF and emphysema has major effects on measures of physiologic function, exercise capacity and prognosis. The composite physiologic index (CPI) was derived to capture the effect of emphysema on IPF. CPI is simple to calculate, **CPI = 91 - (0.65 percent predicted DLCO) - (0.53 percent predicted FVC) + (0.34 percent predicted FEV_1_)**, and has been shown to reflect the extent of disease more accurately than single physiologic indices [38]. CPI is also a powerful predictor of mortality.

### Lung cancer and IPF

An association between IPF and lung cancer was theorized based upon the simultaneous finding of IPF and lung cancer in autopsy studies dating back several decades [40]. A small number of epidemiologic reports helped advance the notion that IPF is an independent risk factor for lung cancer [41,42]. Yet, studies examining multiple causes of death, utilizing information obtained from death certificates, failed to confirm an association between pulmonary fibrosis and lung cancer [43]. A retrospective case-control study took advantage of the British general-practice database and identified 890 cases of IPF. Compared to 5,884 controls, a seven-fold increase in lung cancer was observed in IPF patients [44]. Unsettled issues regarding the association between fibrosis and lung cancer include questions regarding the underlying mechanism of this association; as well as questions regarding the difference in cancer subtype and location in patients with pulmonary fibrosis as compared to the general population [45].

## Etiology and pathogenesis

### Etiology

The term "idiopathic" suggests there are no known causes for IPF. Diagnostic criteria for IPF require exclusion of *known *causes of interstitial lung disease. However, an environmental etiology for IPF is supported by evidence from several sources [46]. A relationship between environmental exposures and IPF is plausible, has been consistently demonstrated by case-control studies and is analogous to known disease, such as asbestosis, in which environmental material is associated with pulmonary fibrosis. Technical obstacles to epidemiologic research have prevented the definitive determination of a causal link between environmental exposure and IPF. Research in this area is hampered by the low prevalence of IPF. Case-control studies, though convenient, are flawed due to selection bias and recall bias.

Meanwhile, cigarette smoking is consistently associated with IPF. A recent study of familial pulmonary fibrosis looked at 309 affected individuals [16]. After adjusting for age and sex, this cohort demonstrated a strong association between smoking and IPF (odds ratio [OR], 3.6; 95% confidence interval [CI], 1.3–9.8).

A multi-center case-control study conducted in the United States included 248 patients with IPF and 491 matched control subjects [47]. This study demonstrated significant associations between IPF and a) cigarette smoking (OR, 1.6; 95% CI, 1.1–2.4); b) silica exposure (OR, 3.9; 95% CI, 1.2–12.7); and c) exposure to livestock (OR, 2.7; 95% CI, 1.3–5.5). Other associations failed to reach statistical significance.

Several intriguing reports suggest the involvement of herpesvirus and/or hepatitis C virus in the etiology of IPF [48-50]. However, another study demonstrated viral infection limited to IPF patients receiving corticosteroids, suggesting that infection is simply a marker of immunosuppression rather than an etiologic agent of fibrosis [51].

### Pathogenesis

While pathogenetic mechanisms are incompletely understood, the currently accepted paradigm proposes that injury to the alveolar epithelium is followed by a burst of pro-inflammatory and fibroproliferative mediators that invoke responses associated with normal tissue repair. For unclear reasons, these repair processes never resolve and progressive fibrosis ensues. This theory is aligned with the most recent scientific data and has been summarized elsewhere [52-54]. A thorough appraisal of the scientific evidence is beyond the scope of this review. However, a few recent advances are worth mentioning.

The origin of pathological fibroblast foci within the IPF lesion remains puzzling. Possibilities include differentiation of resident fibroblasts, recruitment of circulating fibroblast precursors and transdifferentiation of epithelial cells into pathological fibroblast phenotypes.

An animal model has demonstrated bone marrow-derived cells assuming a fibroblastic phenotype and migrating to the lung in substantial numbers following alveolar epithelial cell injury [55]. A separate group of researchers observed migration of fibrocytes to the lungs of animals in a model of epithelial injury [56]. Fibrocytes are circulating cells of hematopoietic origin which are thought to play a role in normal and pathological wound repair [57]. Fibrocytes were previously identified in a variety of fibrotic lesions, including hypertrophic dermal scars and abnormal airways in asthmatics [57].

Transdifferentiation of epithelial cells to a mesenchymal phenotype is a well documented phenomenon that takes place during embryogenesis. Epithelial-to-mesenchymal transition (EMT) is a similar process which has recently been demonstrated as an important pathway mediating fibroproliferation in certain renal diseases [58]. Pulmonary researchers have now demonstrated coexpression of epithelial and mesenchymal markers in histologic specimens obtained from patients with IPF, suggesting a role for EMT in pulmonary fibrosis [59]. In addition, animal models of pulmonary fibrosis demonstrate the possibility of EMT in the lung. One study employed a beta-galactosidase reporter cell to trace epithelial cell lineage during the development of experimentally-induced fibrosis. Mesenchymal markers were noted almost exclusively in cells of epithelial lineage [60].

An overlooked feature of pulmonary fibrosis is the presence of increased angiogenic activity, reminiscent of tumorigenesis. This has been well-established in both animal and human studies [61,62]. An imbalance between angiogenic chemokines (IL-8 and ENA-78) and angiostatic chemokines (IP-10) has been proposed to explain angiogenesis in the development of progressive pulmonary fibrosis [63].

## Diagnostic methods and criteria

### Radiographic findings

**Figure 1:**
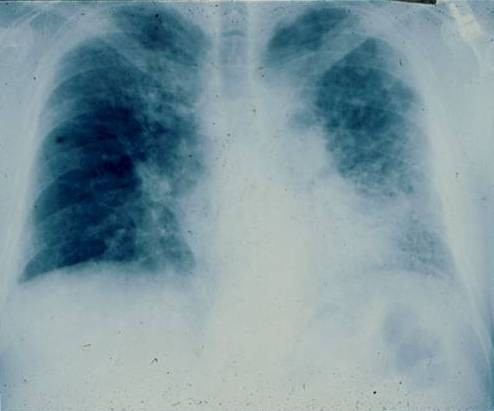
PA chest radiograph of a 67-year old man with progressive dyspnea revealing bilateral reticular infiltrates with lower lobe predominance. * Reprinted from **Fishman's Pulmonary Diseases and Disorders, 4^th ^edition **2007. Meltzer, EB and Noble, PW: Chapter 70, Idiopathic Pulmonary Fibrosis. **With permission from McGraw-Hill Companies**.

The chest roentograph is abnormal in most patients with IPF (Figure 1). Nevertheless, approximately ten percent of patients with histologically proven IPF have a normal roentograph. In these cases, high-resolution computed tomography (HRCT) will reveal evidence of the disease that has been missed by a plain roentograph [1].

Roentographic images of IPF can demonstrate reticular markings (net-like curvilinear opacities). These markings are distributed in a bilateral, asymmetric pattern with predilection toward the lower lobes. A particular pattern of course reticular lines juxtaposed between areas of focal round translucency is known as honeycombing. Roentographic honeycombing emerges late in the course of disease and portends poor prognosis [64].

Use of standard roentographs lacks diagnostic accuracy. A correct diagnosis of IPF (true positive) is made by a roentograph in less than 50% of cases. Furthermore, the radiographic interpretation of interstitial patterns has the characteristic of poor interobserver agreement. Studies examining this particular test characteristic have reported meager 70% concordance amongst radiologists [65,66].

The development of HRCT revolutionized the diagnostic evaluation of interstitial lung disease. HRCT utilizes x-ray technology, along with computerized algorithms, to construct images of virtual thin axial slices through the chest. These high-fidelity images allow a detailed examination of the pulmonary parenchyma. Subsequently, interobserver agreement and overall diagnostic accuracy has been improved with this technology. HRCT permits identification of alternate patterns of diseases. The primary role of HRCT during the diagnostic evaluation of interstitial lung diseases has become the discrimination of "typical" radiographic IPF from that of other ILD.

**Figure 2:**
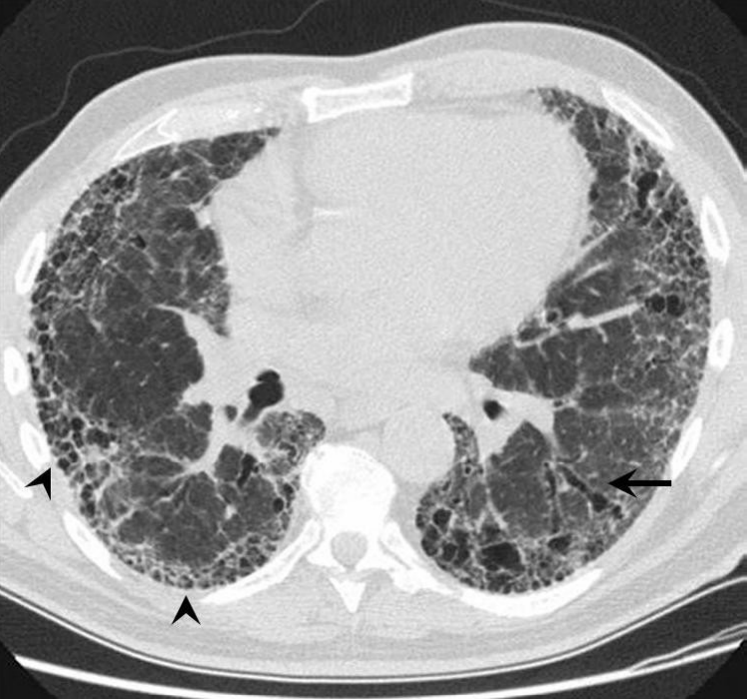
Computed tomography scan illustrates the "classic" features of IPF. Bilateral, peripheral, subpleural reticular infiltrates are evident. The presence of advanced fibrosis is indicated by honeycomb changes (arrowhead) and traction bronchiectasis (arrow). These features permit experienced clinicians to make a confident radiographic diagnosis of IPF. * Reprinted from **Fishman's Pulmonary Diseases and Disorders, 4^th ^edition **2007. Meltzer, EB and Noble, PW: Chapter 70, Idiopathic Pulmonary Fibrosis. **With permission from McGraw-Hill Companies**.

The "typical" appearance of IPF on HRCT consists of patchy, predominantly peripheral, predominantly subpleural and necessarily bibasilar reticular opacification (Figure 2). Ground glass infiltrates can occupy no more than scant, limited areas of the images. Regions of dense reticulation may demonstrate secondary involvement of medium-sized airways that is known as "traction bronchiectasis". The presence of subpleural honeycombing (defined on HRCT as palisades of small, round translucencies), traction bronchiectasis and thickened interlobular septae increases specificity for a diagnosis of IPF. Together, these findings constitute a radiographic pattern that is termed "confident" or "certain" IPF [67].

Certain characteristics need be absent from HRCT in order to label the images as consistent with the "confident" IPF pattern. The HRCT cannot feature predominant ground glass opacities, nodular infiltrates or significant lymphadenopathy. A predominance of upper lobe infiltrates is also inconsistent with "confident" IPF. These findings may suggest alternative diagnoses. For example, ground glass implies heart failure, non-specific interstitial pneumonia, cryptogenic organizing pneumonia, desquamative interstitial pneumonia, respiratory bronchiolitis-associated interstitial lung disease or hypersensitivity pneumonitis. A pattern of fine nodules might suggest hypersensitivity pneumonitis, granulomatous infection or lymphangitic spread of malignancy. Upper lobe disease is found in pulmonary Langerhans' cell histiocytosis, hypersensitivity pneumonitis, several pneumoconioses, sarcoidosis, ankylosing spondylitis, rheumatoid nodules and eosinophilic pneumonia syndromes. Significant hilar lymphadenopathy is associated with sarcoidosis, infection and malignancy.

Several studies examined the accuracy of HRCT utilizing histopathology as the "gold standard" [68,69]. Studies have demonstrated specificity exceeds 90% for the "confident" HRCT pattern. Thus in the right clinical setting, it is possible to make the diagnosis of IPF by HRCT alone. In such cases HRCT obviates the need for lung biopsy.

**Figure 3:**
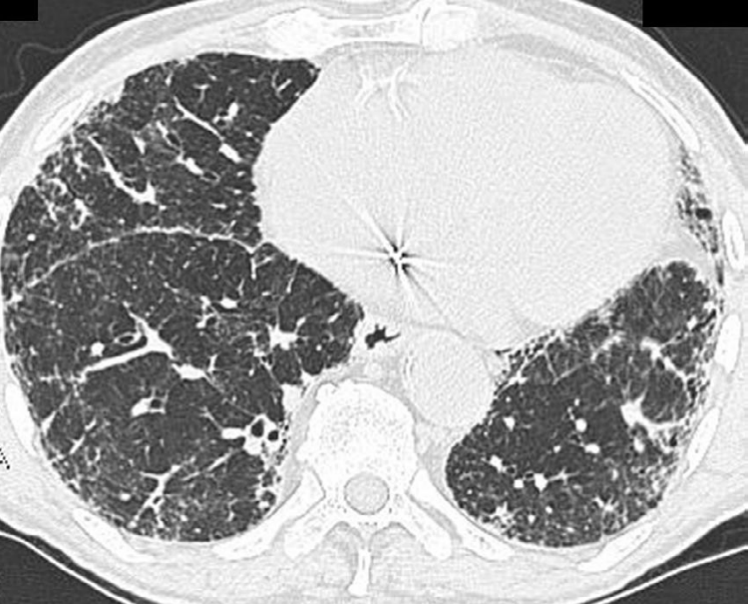
Computed tomography scan of an 81-year old man with biopsy-proven IPF. A peripheral distribution of reticular opacities is demonstrated. Honeycombing and traction bronchiectasis are notably absent. In the absence of specific findings, a surgical lung biopsy was needed to make a diagnosis. * Reprinted from **Fishman's Pulmonary Diseases and Disorders, 4^th ^edition **2007. Meltzer, EB and Noble, PW: Chapter 70, Idiopathic Pulmonary Fibrosis. **With permission from McGraw-Hill Companies**.

However, testing for the "confident" HRCT pattern is not a sensitive tool for case finding of IPF. The full spectrum of the "confident" HRCT pattern can only be found in 4-out-of-5 cases of biopsy-proven IPF. In other biopsy-proven IPF cases, a less specific reticular pattern is seen on HRCT which has been called "possible" IPF (Figure 3). The radiographic pattern of "possible" IPF requires surgical lung biopsy to confirm the diagnosis. Sometimes biopsy identifies an alternative diagnosis.

### Findings of bronchoscopy and surgical lung biopsy

The role of bronchoalveolar lavage (BAL) in the diagnosis of IPF remains limited. While the cell count of BAL fluid from patients with IPF has an expected differential distribution (increased numbers of neutrophils and/or eosinophils), the diagnosis of IPF can not made solely on the basis of BAL fluid analysis. Though much effort has been invested in evaluating the clinical utility of BAL, the results of many studies are contradictory [70,71]. Still, BAL fluid analysis, and sometimes transbronchial biopsy (TBB), can be helpful in excluding alternative diagnoses. Bronchoscopic exam may demonstrate tumor, infection, Langerhans' cells or occupational dust exposures.

A surgical lung biopsy is close to the "gold standard" for diagnosis and is recommended to confirm all suspected cases of IPF. Biopsy from two sites is recommended based on data indicating a substantial risk of sampling era unless specific effort is made to reflect the gamut of gross disease [72].

In cases presenting with a "confident" HRCT pattern, biopsy can be avoided because the results of biopsy can be predicted [68,69]. A sizable tissue specimen is required in order to distinguish patterns of IIP, one from the other. Therefore, surgical biopsy is needed and transbronchial biopsy is inadequate. A surgical lung biopsy can be performed by either open thoracotomy or a video-assisted thoracoscopy (VATS) approach. VATS is preferred since this procedure is associated with lower morbidity and shorter hospital stay as compared with open thoracotomy [73].

The decision to perform surgical lung biopsy must be carefully considered. Advanced lung disease, poor functional status and older age are relative contraindications to surgery. The absolute risk associated with biopsy is controversial and all of the evidence related to this issue is derived from retrospective data, thus colored by inherent selection bias. While some studies have noted a high short-term mortality, other studies have demonstrated that surgical lung biopsy can be performed safely [74,75]. VATS is usually well tolerated and can provide useful information concerning the diagnosis, prognosis and treatment options.

**Figure 4:**
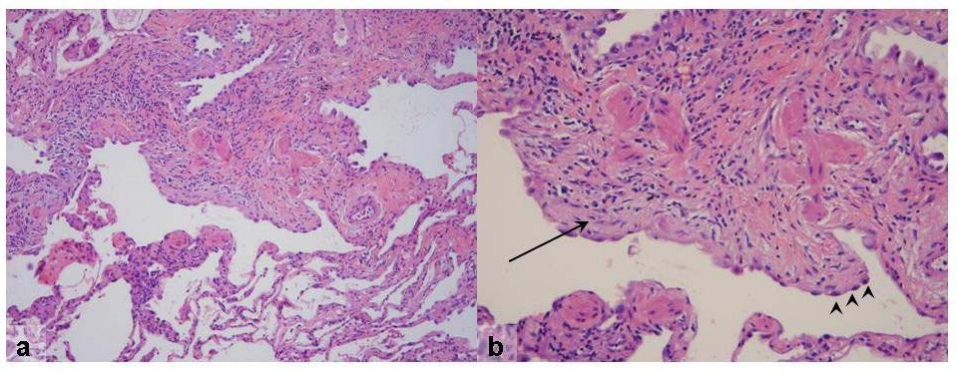
**a) **Low-magnification photomicrograph of UIP showing the characteristic heterogeneous involvement of the parenchyma. Zones of interstitial fibrosis are seen alternating with areas of normal lung. **b) **Higher-magnification demonstrates enlarged cystic airspaces lined with hyperplastic alveolar epithelium (arrowheads). Beneath the mucosal layer is an advancing region of young fibrosis containing loose extracellular matrix (pale pink staining) and fibroblasts (arrows). * Reprinted from **Fishman's Pulmonary Diseases and Disorders, 4^th ^edition **2007. Meltzer, EB and Noble, PW: Chapter 70, Idiopathic Pulmonary Fibrosis. **With permission from McGraw-Hill Companies**.

**Figure 5:**
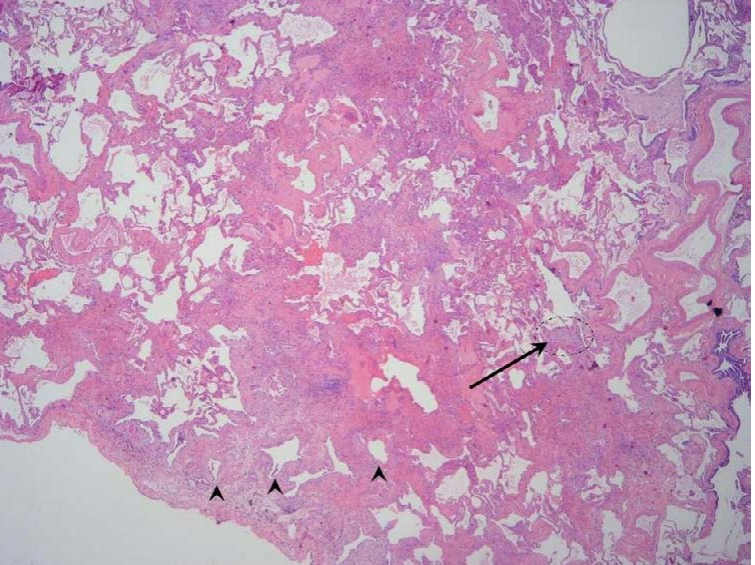
Scanning view of UIP demonstrates the characteristic variegated appearance of UIP. Note the honeycomb change (arrowheads) present in the region of dense fibrosis adjacent to the pleural surface. A fibroblast focus (arrow) is seen at the leading edge of advancing fibrosis. * Reprinted from **Fishman's Pulmonary Diseases and Disorders, 4^th ^edition **2007. Meltzer, EB and Noble, PW: Chapter 70, Idiopathic Pulmonary Fibrosis. **With permission from McGraw-Hill Companies**.

The gross pathology of IPF may be normal, but often a distinctive nodular appearance of the pleural surface is found. This has been likened to cirrhosis of the liver. The histopathological lesion associated with IPF is known as usual interstitial pneumonia (UIP). This lesion is defined by a distinctly variegated pattern. UIP features normal lung architecture alternating with patchy areas of histologically apparent pulmonary parenchymal fibrosis (Figures 4 and 5). Fibrosis takes the form of alveolar septal thickening with marked involvement of the subpleural regions. The most severely involved areas of the lung demonstrate complete distortion of normal architecture, with sheets of dense collagen replacing normal lung tissue and occasional cystic structures known as microscopic honeycombs. When examined carefully under the microscope, the region of scarred lung tissue appears to encroach upon areas of preserved, normal lung tissue. This has been termed the "leading edge" of fibrosis and contains specialized structures known as fibroblast foci. Fibroblast foci are pale-staining whirls of loose extracellular matrix molecules, interspersed with numerous cells of the fibroblast type (Figures 4 and 5). Inflammation is mostly absent from the UIP pathologic pattern except for occasional lymphoid follicles that are confined to regions of end-stage fibrosis. UIP contains no hyaline membranes, granulomas or organized alveolar exudates. Sometimes emphysema or respiratory bronchiolitis is superimposed upon the UIP pattern when the patient is a former or active smoker. These pathological changes can complicate diagnostic interpretation.

It is important to note that the UIP pattern is found in several diseases and is not limited to IPF. UIP can be associated with connective tissue disease, asbestosis, hypersensitivity pneumonitis, the Hermansky-Pudlak syndrome and drug toxicities (*e.g*.: bleomycin, amiodarone and nitrofurantoin toxicity). Distinguishing IPF from other disorders that contain UIP requires correlation with the clinical history.

It is also important to realize that honeycomb change of itself is a non-specific manifestation of end-stage fibrosis. Microscopic honeycombs do not equate with the UIP pattern nor do they connote a diagnosis of IPF. Only the full spectrum of UIP is diagnostic for IPF (in the correct clinical setting, as noted above).

### Diagnostic criteria

The actual "gold standard" diagnosis of IPF consists of clinical-radiological-pathological correlation and was defined by consensus conference in the year 2000 and adopted by the American Thoracic and European Respiratory Societies (ATS/ERS) in a statement of guidelines published in the same year [1]. According to guidelines, the diagnosis of IPF can be considered definitive only in the presence of a surgical (not transbronchial) lung biopsy.

The **definite diagnosis **of IPF requires all of the following: [1]

• Surgical lung biopsy revealing a histologic pattern consistent with UIP

• Exclusion of other known causes of interstitial lung disease (*e.g*.: connective tissue disease, environmental exposure, *etc*.)

• Abnormal pulmonary physiology with evidence of restriction and/or impaired gas exchange (can exist during exercise alone)

• HRCT demonstrating a pattern of "confident" or "possible" IPF.

In the absence of a surgical biopsy, the diagnosis of IPF remains uncertain. Yet, a set of reproducible clinical criteria were developed to define the **probable diagnosis **of IPF in cases in which a surgical biopsy is not possible. These clinical criteria were endorsed by the ATS/ERS consensus statement on IPF [1]. By consensus opinion, IPF can be reasonably diagnosed if all four major criteria and three-out-of-four minor criteria are satisfied. They are as follows:

**Major criteria **(Must fulfill all four requirements)

• Exclusion of other known causes for interstitial lung disease (such as drug toxicity, environmental exposure and connective tissue disease)

• Abnormal pulmonary function testing that includes evidence of restriction (reduced VC often with an increased FEV_1_/FVC ratio)and/or impaired gas exchange (increased A-a gradient or decreased diffusion capacity)

• Bibasilar reticular abnormalities with minimal ground glass opacities on HRCT scans (a "confident" HRCT is preferred)

• Transbronchial lung biopsy or bronchoalveolar lavage (BAL) does not support an alternative diagnosis

**Minor criteria **(Must fulfill at least three; in addition to major criteria)

• Age > 50 yr

• Insidious onset of otherwise unexplained dyspnea on exertion

• Duration of illness ≥ 3 months

• Bibasilar, inspiratory crackles (dry or "Velcro" type in quality)

These criteria have never been subjected to a prospective analysis and, over time, diagnostic algorithms have continued to evolve. As HRCT technology has improved and the utility of this modality has been consistently demonstrated in clinical trials, HRCT has become a more important tool in diagnostic algorithms. Meanwhile, transbronchial biopsy and BAL have fallen from favor, mostly due to low diagnostic yield. A new ATS/ERS-sponsored consensus statement should address these issues and publication of such is expected in 2008.

### Controversies regarding the diagnosis of IPF

The clinical-radiological-pathological "gold standard" diagnosis of IPF is flawed due to several issues, such as: 1) lack of standardized tests to exclude known causes of interstitial lung disease; 2) poor interobserver agreement regarding the interpretation of radiographic images; and, 3) poor interobserver agreement as regards the recognition of histological patterns. Interobserver agreement amongst radiologists reading HRCT has been reported to be no better than 80% [76]. Agreement amongst pathologists has been shown to depend upon experience and training and can be as low as 50% [77,78].

Since the mid-1990s, attention has focused on the division of idiopathic pulmonary fibrosis into histologically-defined subgroups. This practice stems from the description of NSIP in 1994 [79]. NSIP pathology consists of homogeneously thickened interstitial spaces that contain accumulated fibrosis and inflammation. In NSIP, fibroblastic foci are scarce and focal areas of organizing pneumonia can be found but remain inconspicuous. It has been suggested that patients with NSIP live longer than patients whose biopsy contains UIP [9]. However, a clinical description of NSIP-associated symptoms, along with vital statistics and risk factors for NSIP, have never been systematically recorded. By default, NSIP has come to represent a disease that exists in parallel to IPF. Whether NSIP truly represents a separate disease from IPF remains to be shown. Patients with NSIP are usually ten years younger than those with UIP. NSIP is also reported to be more sensitive to corticosteroids [2]. Some authors suggest that NSIP represents an early stage of IPF but this issue is highly controversial [54,80].

Three lines of evidence suggest that NSIP and UIP are different ends of a spectrum resulting from the same disease. The first piece of evidence is found in the examination of patients undergoing multiple surgical lung biopsies. One such study found that 26% of patients with IPF have NSIP pathology in one lobe while simultaneously displaying UIP in a sample from another lobe [72]. The second piece of evidence is provided by a study that examined survival, histopathology and pulmonary functions trends [5]. This study compared a cohort with 12-month interval physiologic stability to a cohort with declining 12-month physiology. It was found that physiology predicts mortality more strongly than any other measurable parameter, including histopathological distinction (*i.e*.: UIP *vs*. NSIP); in fact, pathological pattern conferred no independent prognostic value. The last bit of evidence comes from a cohort of families affected by familial pulmonary fibrosis where both NSIP and UIP were often found within a single family [16].

## Differential diagnosis

The differential diagnosis of IPF includes other idiopathic interstitial pneumonias. HRCT is useful for excluding disease with predominantly ground glass opacity or nodular patterns. Non-specific interstitial pneumonia (NSIP) will always remain in the differential and, in some cases, can only be excluded by biopsy.

Connective tissue diseases such as systemic sclerosis, polymyositis or rheumatoid arthritis can mimic IPF, both clinically and radiographically. The great majority of patients with systemic sclerosis will present with HRCT scan features more closely resembling NSIP [81]. Yet, remember that such an "atypical" HRCT pattern does not exclude IPF (see the discussion of HRCT patterns above). Elicitation of specific symptoms and the measurement of autoantibodies can distinguish these entities from IPF.

There are also *forme fruste *autoimmune disorders which can be difficult to recognize. These entities comprise autoimmune-mediated lung disease without a constellation of signs and symptoms to fulfill diagnostic criteria for defined rheumatologic illness. The presence of one or more symptoms, such as Raynaud's phenomenon, proximal muscle weakness or sicca features, coupled with laboratory features of systemic inflammation (antinuclear and other specific autoantibodies) define the syndrome of undifferentiated connective tissue disease that can accompany pulmonary fibrosis and resemble IPF [82].

Chronic hypersensitivity pneumonitis and other environmental (sometimes occupational) exposures can also be difficult to differentiate. The clinical history can serve to discriminate this condition but is oftentimes equivocal. Nonetheless, a history of exposure to asbestos, grain dust and mold should be sought during the initial evaluation of IPF. Radiation pneumonitis, end-stage sarcoidosis, certain drug toxicities (*e.g*.: bleomycin, nitrofurantoin, amiodarone, carmustine and methotrexate) and several congenital disorders (*e.g*.: Hermansky-Pudlak, Gaucher's disease, Niemann-Pick disease and dyskeratosis congenital) are also in the differential.

## Management

Current medical therapy for IPF is poorly effective, at best. However, IPF is a progressive, ultimately fatal disorder for which substantive medical therapy is desperately needed. This has led to a history of undue excitement over novel treatment modalities and, sometimes, new medications have been adopted prematurely only to loose credibility with further study. At present, expectant management is the most reasonable approach to IPF care along with supportive measures instituted as necessary. Patients who satisfy enrollment criteria can be enlisted in clinical trials to test novel medications that may prove useful in the future. In addition, patients with IPF can be offered lung transplantation if they are below the age of 70 years. The timing for lung transplantation depends upon an accurate assessment of risks and benefits. This begins with an assessment of disease activity and prognosis in IPF.

### Prognostic indicators

Early studies looking at the prognosis of IPF identified older age, male sex, significant dyspnea, severe physiologic abnormalities, advanced fibrosis and a poor response to therapy as factors predicting shortened survival [83]. Early studies were limited by the use of retrospective study designs.

Recently, systematic studies have evaluated specific features of IPF that are predictive of mortality. Features of the surgical biopsy specimen have been evaluated and it was found that neither the degree of cellularity nor degree of fibrosis could predict survival [84]. However, the presence of "young" connective tissue, characterized by multiple fibroblast foci, was found to correlate with shortened survival. Other investigators have confirmed this link between fibroblast foci and mortality [85]. It was also found that the extent of fibroblast foci can predict physiologic functions such as vital capacity and diffusion capacity.

Three separate groups of investigators observed a relationship between physiologic measures and survival amongst well-defined cohorts of IPF patients [3-5]. Of particular interest, one study reported that physiologic measures were more accurate than histopathology (NSIP *vs*. UIP) in predicting mortality [5]. Twelve-month trends in diffusion capacity were shown to predict survival. In this study, physiologic measures were taken at baseline and at a twelve month follow-up visit. The patients were grouped into two categories, demonstrating significant decline (more than 15% of baseline) versus demonstration of stability or improvement. Mortality was shown to be substantially higher in the group demonstrating decline.

Other investigators have examined IPF patients and identified interval decline of forced vital capacity as a characteristic that is predictive of survival. A 10% decline of forced vital capacity, at either six or twelve months, had poor prognostic implications and was more accurate than predictions based upon baseline physiologic parameters alone [3].

The ability of HRCT to predict the outcome of IPF was also demonstrated. When biopsy-proven IPF patients were followed for three years by HRCT, it was found that radiographic honeycombing predicted the worst survival [86]. In addition, when radiographic fibrosis and histopathologic fibrosis were assigned scores, they were found to be equivalent with respect to predicting death or clinical worsening.

Another interesting study examined the relationship between the "confident" IPF pattern and survival. A cohort of patients with biopsy-proven IPF were analyzed by HRCT and divided into groups based upon radiographic/pathologic concordance. UIP pathology was shown to confer a worse prognosis when seen in combination with the "confident" IPF pattern. It was found that the an "indeterminate" pattern of HRCT conferred better prognosis despite the presence of UIP on biopsy [87].

### Pharmacotherapy

In the past, unremitting inflammation was thought to cause progressive pulmonary fibrosis. Therefore treatment regimens were designed to suppress the immune system with the goal of halting subsequent fibroproliferation. However, large randomized and placebo-controlled trials were never performed to assess the efficacy of this strategy. The only evidence in support of immunosuppressive therapy for IPF is a handful of small studies. Nonetheless, the ATS/ERS consensus statement recommends the use of corticosteroids combined with a cytotoxic agent for carefully selected IPF patients [1]. The consensus statement recommends prednisone (starting at 0.5 mg/kg and tapered to a maintenance level of 0.125 mg/kg), combined with either azathioprine or cyclophosphamide (the dose targeted to 2–3 mg/kg). Combination therapy is suggested for a period of at least six months with clinical and physiological response used to guide further management.

The best evidence in support of the prednisone/azathioprine regimen comes from a prospective, randomized, double-blind study of only 27 IPF patients [88]. This study examined survival and lung function over a period of several years. While no statistically significant difference was measured using unadjusted data at the end of the first year, a marginal advantage to the prednisone/azathioprine regimen was demonstrated by examining age-adjusted survival curves. This study was performed in the era prior to the initial description of NSIP, which has a better prognosis than IPF. It is most probable that some of the 27 cases included in this study were, actually, unrecognized cases of NSIP. Further inspection of the survival curves from this study reveal that the difference in survival occurs after the fourth year of treatment. Since most IPF patients die within the first three years following diagnosis, such a late treatment benefit would be useless.

A single randomized study of 43 IPF patients compared prednisolone alone to prednisolone/cyclophosphamide [89]. This study followed lung function, radiographic images, dyspnea scores and survival. While the study could not demonstrate a survival benefit from combination therapy, an analysis of time to treatment failure was favorable toward the prednisone/cyclophosphamide group. Because this study was also performed in the era before NSIP, its results are confounded by possible inclusion of cases other than IPF.

Two recent studies re-evaluated the utility of prednisone/cyclophosphamide in the treatment of IPF but both studies failed to support its use [90,91]. The first study followed a series of 19 IPF patients, treated with cyclophosphamide following a corticosteroid taper. This study contained no control group and only one patient was shown to stabilize while undergoing cytotoxic therapy. The study concluded that cyclophosphamide conferred no benefit in the treatment of IPF. The other study utilized a retrospective design to evaluate survival time amongst 82 IPF patients receiving prednisone/cyclophosphamide. The IPF patients were compared to an untreated age-matched, lung-function matched cohort that served as the control group. No survival benefit was observed. Overall, given its considerable toxicity and lack of support for efficacy, it seems unreasonable to prescribe prednisone/cyclophosphamide for the treatment of IPF.

Another potential therapy for IPF is N-acetylcysteine (NAC) which is a molecular precursor to the naturally occurring antioxidant glutathione. Glutathione is known to be depleted in the lungs of patients with IPF [92]. Theoretically, oral NAC should replete glutathione stores and restore natural oxidant/anti-oxidant balance to prevent oxidative injury that precedes fibroproliferation [93]. A small prospective, but uncontrolled, study following 18 IPF patients receiving NAC demonstrated restoration of glutathione levels and improvement of lung function measures [94]. This pilot study prompted a large, randomized, double-blinded study of 182 IPF patients to compare the efficacy of a regimen that included prednisone, azathioprine and NAC to a regimen of only prednisone and azathioprine [23]. A statistically significant difference in lung function was found to favor treatment with prednisone/azathioprine/NAC. However, the study is flawed because of a high rate of drop-out among the study subjects. Drop-out was addressed in statistical analysis by deriving and using imputed data for missing data points. Both groups in the study experienced decline of lung function over the 12-month study period; the difference was that one group declined more. The regimen of prednisone/azathioprine/NAC was shown to be possibly superior to prednisone/azathioprine alone. However, the efficacy of prednisone/azathioprine/NAC has not been compared to placebo and has never been shown to improve or stabilize patients with IPF.

Recently, a panel of experts were asked to rate the evidence for the various treatment options for IPF [95]. The panel concluded that the most appropriate pathway was to enroll eligible patients in clinical trials or refer for lung transplantation as indicated. Patients without access to clinical trials or lung transplantation could be offered other therapy but the sole use of corticosteroids was deemed inappropriate. The use of corticosteroids in conjunction with azathioprine was deemed acceptable. Given the evidence for prednisone/azathioprine/NAC, this regimen could be considered with little risk attributable to NAC.

Several clinical trials are presently assessing the utility of novel agents in the treatment of IPF. One promising drug is pirfenidone which has already been tested in phase I and phase II studies in the United States and Japan. A study examining the use of pirfenidone enrolled 105 IPF patients to receive either the study drug or placebo [21]. The primary endpoint of this study was gas exchange as measured by pulse oximetry during a six-minute walk. This study was discontinued prematurely due to concerns over excess mortality in the placebo group. Analysis revealed no difference between groups when assessed for the primary endpoint. However, pirfenidone was shown to confer benefit in measures of forced vital capacity and survival.

A word of caution: experience has shown that, although a drug may appear promising in small phase II trials, large trials with additional power to determine efficacy may, in fact, reveal that a drug is ineffective. This was recently demonstrated by trials investigating the medication interferon-**γ **(IFN-**γ**). In the first multicenter study, 330 patients with IPF were randomized to receive either IFN-**γ **or placebo [22]. Patients were treated for 48 weeks with study drug and the primary endpoint measured was the effect on progression-free survival (a composite measure that included death and physiologic decline). The study showed no benefit from IFN-**γ **as measured by the primary endpoint. However, analysis of secondary endpoints revealed a trend toward improved survival in the group receiving IFN-**γ**. This trend did not reach statistical significance (p = 0.08), but the study was not powered to detect an effect on survival. Therefore, a second trial was designed to specifically evaluate survival, with a plan to enroll over 800 IPF patients. Unfortunately, this second trial was recently discontinued after a planned interim analysis determined a lack of benefit from IFN-**γ **relative to placebo (unpublished data).

### Non-pharmacological treatments

#### Lung transplantation

A survival benefit has been demonstrated for lung transplantation in IPF patients [96]. However, transplantation is only appropriate for carefully selected patients. Currently, five-year post-transplant survival approaches 50% [97]. Rejection remains a common and formidable problem leading to significant post-transplant morbidity and mortality. Following transplantation, patients require lifelong treatment with a combination of immunosuppressants in order to prevent rejection. Patients must also submit to frequent surveillance bronchoscopy, for the purpose of identifying infectious and inflammatory complications.

The timing of pulmonary transplantation poses additional challenges. Until recently, early referral was advocated for all patients with IPF because of long pre-transplant waiting times exceeding the median survival time of patients with IPF. However, new allocation scores in the U.S., devised to alleviate transplant waiting list mortality, have dramatically reduced waiting times for patients with IPF thus removing the impetus for early referral [98]. Now the decision to refer a patient for transplant revolves around the identification of the small subgroup of patients with IPF that might survive longer without transplant [96]. The judicious use of prognostic indicators, as discussed above, can inform such judgment.

The decision to perform lung transplantation in a patient with IPF requires careful consideration of the risks and benefits of such an undertaking. Advanced age precludes many patients with IPF from serious consideration of lung transplantation. Lung transplantation should be reserved for those with adequate social support and limited comorbidities, in order to face the rigors of post-transplantation medical management. Lung transplantation, on the whole, is best performed at specialized centers that employ experienced surgeons and physicians who are familiar with post-transplantation management.

#### Supportive measures

Regardless of the primary therapy, patients with IPF need to be treated with supportive measures as clinically indicated by their condition. For example, exercise-induced hypoxemia warrants a prescription for supplemental oxygen. While supplemental oxygen has been shown to improve exercise performance in chronic obstructive pulmonary disease (COPD), it has not been rigorously evaluated for the treatment of IPF. Nevertheless, a study that examined quality of life (QOL) in patients with IPF found no difference in QOL between patients receiving supplemental oxygen compared with those not receiving oxygen [99]. This is in spite of the fact that patients requiring oxygen were sicker.

Patients with IPF should be encouraged to enroll in a program for pulmonary rehabilitation. Pulmonary rehabilitation has not been rigorously examined in IPF, though quadriceps strength has been correlated with exercise capacity amongst patients with IPF [100]. This implies that training of the lower extremities could increase exercise capacity of IPF patients, as it does for patients with COPD. Because overall QOL is impaired in IPF, with specific deficits in the areas of physical health and perceived independence, it is reasonable to assume that rehabilitation programs, designed to increase physical well being and independence, will improve QOL [99].

## Unresolved issues

IPF remains a disease for which the etiology is unknown. The pathogenesis is only poorly understood and the natural history of the disease is just beginning to reveal itself through observation of placebo groups from several large multi-center clinical trials. There is no definitive approach to the treatment of IPF because evidence for effective medical therapy is still lacking. Future directions for research should include programs that encourage the search for new molecular targets for therapy; and research to identify genetic susceptibility factors [101,102]. Several centers are banking tissues from IPF patients that will enable translational research in the field. A multicenter clinical network, sponsored by the United States' National Institute of Health, was recently established to facilitate the study of novel therapeutic agents as appropriate. In the next decade, it is likely that considerable progress will be made toward understanding and treating this devastating illness.
